# The non-human reservoirs of Ross River virus: a systematic review of the evidence

**DOI:** 10.1186/s13071-018-2733-8

**Published:** 2018-03-19

**Authors:** Eloise B. Stephenson, Alison J. Peel, Simon A. Reid, Cassie C. Jansen, Hamish McCallum

**Affiliations:** 1 Environmental Futures Research Institute, Griffith University, Brisbane, Queensland 4111 Australia; 2 The University of Queensland, School of Public Health, Herston, Brisbane, Queensland 4006 Australia; 3 Metro North Public Health Unit, Metro North Hospital and Health Service, Windsor, Brisbane, Queensland 4030 Australia; 4 Communicable Diseases Branch, Department of Health, Queensland Government, Herston, Brisbane, Queensland 4006 Australia

**Keywords:** Amplifier, Experimental infection, Serology, Virus isolation, Host, Vector-borne disease, Arbovirus

## Abstract

Understanding the non-human reservoirs of zoonotic pathogens is critical for effective disease control, but identifying the relative contributions of the various reservoirs of multi-host pathogens is challenging. For Ross River virus (RRV), knowledge of the transmission dynamics, in particular the role of non-human species, is important. In Australia, RRV accounts for the highest number of human mosquito-borne virus infections. The long held dogma that marsupials are better reservoirs than placental mammals, which are better reservoirs than birds, deserves critical review. We present a review of 50 years of evidence on non-human reservoirs of RRV, which includes experimental infection studies, virus isolation studies and serosurveys. We find that whilst marsupials are competent reservoirs of RRV, there is potential for placental mammals and birds to contribute to transmission dynamics. However, the role of these animals as reservoirs of RRV remains unclear due to fragmented evidence and sampling bias. Future investigations of RRV reservoirs should focus on quantifying complex transmission dynamics across environments.

**Electronic supplementary material:**

The online version of this article (10.1186/s13071-018-2733-8) contains supplementary material, which is available to authorized users.

## Background

### Vertebrate reservoir hosts

Globally, most pathogens of medical and veterinary importance can infect multiple host species [1]. Indeed, an estimated 60–75% of emerging infectious diseases are multi-host zoonoses [2]. Zoonotic arboviruses, such as Rift Valley fever virus, West Nile virus, and Japanese encephalitis virus, have complex transmission cycles that include multiple host and vector species in maintenance and spillover [2–4]. Identifying optimal approaches to mitigate spillover of multi-host pathogens requires an understanding of how the transmission cycles of zoonotic viruses and non-human hosts contribute to spatiotemporal changes in the patterns of human disease [1, 5, 6]. The challenge of understanding the complex population biology of multi-host pathogens comes not only from identifying potential reservoir host species, but in disentangling which species contribute most to transmission and pathogen pressure, and whether any species are crucial to persistence within the reservoir community [7, 8].

The definition of a “reservoir” in infectious disease epidemiology is not straightforward [7]. This is especially the case for arboviruses, where complex and novel transmission dynamics among arboviruses has resulted in multiple definitions for the key term “reservoir” [9]. Given the diversity of virus-vector-vertebrate host interactions, there is unlikely to be a single definition suitable for all systems or for all applications [9]. Here, we are concerned with identifying which vertebrate hosts contribute most to the pathogen pressure on humans (*via* infected vectors). We therefore adopt the notion that an arbovirus “reservoir” is a vertebrate host species which, if present in sufficient abundance, will contribute to the pathogen pressure on humans. This will require that it has frequent contact with vector populations, is attractive to a vector as a blood meal source, is susceptible to infection, and can produce sufficient viraemia to infect another vector [9–11]. Kuno and Chang [3] identified three commonly used criteria for classifying vertebrate reservoirs of arboviruses: (i) virus isolation from suspected animals; (ii) relatively high antibody prevalence in the animals captured in the field; and (iii) demonstration of viraemia (of high virus titre and duration) in the suspected animals typically obtained under lab conditions. Methods commonly adopted to address these criteria include virus isolation, serosurveys and experimental infection studies, respectively.

### Ross River virus

Ross River virus (RRV) is a zoonotic alphavirus and human infection is nationally notifiable in Australia. It is responsible for the greatest number of mosquito-borne infections in humans across every state and territory of Australia [12]. On average there are 4800 cases of RRV notified per year Australia-wide, with the majority from Queensland [12]. There are occasional large outbreaks of RRV involving a significantly higher number of human cases. In 2015, there were 9800 notifications of RRV - almost double the national average, 6192 of which were reported from Queensland. In 2017, an outbreak occurred in Victoria, with some 1200 notifications reported in January and February alone, exceeding the state counts for the previous four years combined [12].

Ross River Virus is not usually fatal [13]. However, patients with disease caused by RRV infection present with symptoms that include polyarthritis, myalgia, and fever and chronic joint pain which may last several weeks and, in some cases, months [14–16]. The economic costs of illness were estimated to be A$1070 per case in 2002, averaging more than A$5 million each year [17]. This is likely a conservative estimate of RRV cost as it does not include broader implications of infection, such as the inability for an individual to work or care for children [13]. Presently, there is no treatment or commercially available vaccine for RRV and the best means to reduce the risk of infection is through mosquito management and avoidance of mosquito bites [14, 18].

More than 40 species of mosquitoes have yielded isolates of RRV (summarised in [19]), although many are likely to only have a minor role in transmission. Species most commonly associated with transmission include saltmarsh mosquitoes *Aedes camptorhychus*, presenting in southern Australia and is replaced by *Ae. vigilax* north of its range, and the freshwater mosquito (*Culex annulirostris)* that is present throughout Australia, excluding Tasmania [14].

A definitive description of the host-vector relationships in the transmission cycle of RRV is currently not available. Non-human reservoirs of RRV are thought to play a significant role in RRV endemicity [20–22]. While several authors have suggested that human-mosquito-human transmission of RRV may occur during epidemics [23–25], such transmission is not believed to be sufficient to account for the total number of reported cases each year in Australia [19], nor to be responsible for the long-term persistence of RRV.

Marsupials are generally considered better reservoirs of RRV than placental mammals, which in turn are better reservoirs than birds [13, 19, 26, 27]. This hypothesis first appeared in the literature in 1971 following epidemiological studies in northern Queensland where high rates of RRV seropositivity were detected in macropods (kangaroos and wallabies) [28]. However, the hypothesis deserves critical re-evaluation because there is evidence that RRV circulates in countries in the Pacific, where marsupials are absent [29–31].

This review aims to: (i) critically review the evidence supporting the hypothesis ‘marsupials are better reservoirs of RRV than placental mammals, which in turn are better reservoirs than birds’; (ii) characterise the limitations of that evidence; and (iii) identify research gaps with regards to RRV transmission cycles.

## Methods

We systematically identified original research papers on RRV reservoir as follows. First, we searched electronic databases (Web of Science, ProQuest, Science Direct, PubMed and Google Scholar) for articles published between 1950 and May 2016 using combinations (Additional file 1: Table S1) of the following keywords: ‘Ross River virus’, ‘Ross River fever’, ‘endemic polyarthritis’, ‘host’, ‘reservoir’, ‘wild*’, ‘captive’, ‘population’, ‘serolog*’, ‘serosurvey*’, ‘antibod*’, ‘virus’, ‘viral’, ‘viraemia’, ‘viremia’, ‘PCR’, ‘patholog*’, ‘serum’, ‘RNA’, ‘vector*’. The asterisk (*) operator was used as a wildcard to search for all the possible variations of keywords. We then manually searched bibliographies for additional references. Review papers, studies involving only humans, and studies not reporting original data were excluded. A flow chart showing the article selection process is presented in Additional file 2: Figure S1. A list of the publications included is provided in Additional file 1: Table S2. One person (EBS) was responsible for determining if a paper was included and extracting data. By following the inclusion and exclusion criteria there were no discrepancies for selecting papers.

For each article, we recorded the following information: year of publication, location of study, type of study (experimental infection, serosurvey, virus isolation/detection), method (e.g. for experimental infection studies: the dose, infection technique, strain of RRV used and post infection analysis), species investigated, sample size and results. Species examined in each study were assigned to a species group (marsupial, placental mammal, bird) for interpretation.

### Statistical analysis

A meta-analysis of results across experimental infection studies was not possible as methods of infection and viral detection were highly variable. Instead, we conducted two one-way analysis of variance (ANOVA) for the one experimental infection study that assessed the greatest number of species (*n* = 10 species, [27]) to test the hypothesis that the duration of viraemia and peak viral titre differs between species groups (marsupial, placental mammal and bird).

For serosurveys, the seroprevalence range was calculated for each species group (marsupial, placental mammal or bird), and plotted as a boxplot. An ANOVA was used to test for differences in seroprevalence between species groups across different studies.

## Results

We identified a total of 38 research papers that met our criteria. Of these studies, seven described experimental infections, five described virus isolation and 29 utilised serosurveys (Table 1) (three studies used multiple methods, Additional file 1: Table S2). All experimental infection studies were undertaken in Queensland. Virus isolation studies were undertaken in Queensland and Victoria. We identified a single article that performed molecular identification of virus from horses [32], but this was excluded from the analysis as the paper was methodological, describing the novel test method. Serosurveys were performed in every state in Australia and the Northern Territory, as well as other countries including Fiji (*n* = 1), New Guinea (*n* = 1) and New Zealand (*n* = 2) (Table 1). The earliest studies of RRV reservoirs included serosurveys in 1966 and virus isolation in 1968.

**Table 1:** Summary of study types included in the literature review of Ross River virus reservoir studies comprising the number of studies of each type, location and dates of publications

Study type	Total no. of studies	Location of studies	Date range of studies
Experimental infection	7	Queensland	1969–2001
Virus isolation	5	Queensland, Victoria	1968–2003
Serosurvey	29	Queensland, New South Wales, Western Australia, Victoria, South Australia, Northern Territory, Tasmania, New Guinea, Fiji, New Zealand	1966–2015

### Experimental infection studies

The seven experimental infection studies included infection of 18 vertebrate species with RRV (summarised in Table 2). At least two strains of RRV were used: the prototype T48, isolated from a human Townsville in 1959 [13], and B94/20, isolated from a human during an epidemic in Queensland in 1994 [33]. Two studies did not state which strain was used [27, 34]. The most common route of infection was *via* infected mosquito (*n* = 5), although subcutaneous (*n* = 2) and intravenous (*n* = 1) routes were also used. All studies assessed the titre of viraemia in blood, but methods and metrics differed. Four of the seven studies subsequently exposed infected animals to susceptible vectors to determine infectiousness of potential reservoirs. Across all studies, the median sample size was 9 individuals, Kay et al. [27] using the largest sample size of 20 chickens (*Gallus gallus domesticus*). More than half (4 of 7) of the experimental infection studies undertaken for RRV simultaneously co-infected the same animal with other viruses in addition to RRV, including Barmah Forest virus, Murray Valley encephalitis or Sindbis virus [27, 35–37].

**Table 2:** Summary of Ross River virus experimental infection studies included in the review with description of methods and vertebrate species used

Reference	Infection method			Virus detection methods	Vertebrate species tested (sample size)	Virus co-infection
	RRV strain	Infection route	Vector used			
Boyd et al. [35]	B94/20	Infected vector bite	Yes	Infection of a vector; magnitude and duration of viraemia (SMIC)	Brushtail possum *Trichosurus vulpecula* (10)	Simultaneous infection with Barmah Forest virus
Boyd & Kay [58]	B94/20	Infected vector bite	Yes	Infection of a vector; magnitude and duration of viraemia (CCID_50_)	Cat *Felis catus* (10); dog *Canis lupus familiaris* (10)	
Ryan et al. [33]	B94/20	Infected vector bite	Yes	Infection of a vector; magnitude and duration of viraemia (TCID)	Grey-headed flying fox *Pteropus poliocephalus* (10)	
Kay et al. [34]	Not stated	Intravenous injection and infected vector bite	Yes	Infection of a vector; magnitude and duration of viraemia (SMIC)	Horse *Equus ferus* (11)	Simultaneous infection with Murray Valley encephalitis
Kay et al. [27]	Not stated	Infected vector bite	Yes	Infection of a vector; magnitude and duration of viraemia (SMIC)	Agile wallaby *Macropus agilis* (9); grey kangaroo *Macropus giganteus* (3); rabbit *Oryctolagus cuniculus* (9); sheep *Ovis aries* (8); pig *Sus scrofa* (11); horse *Equus ferus* (11); cattle *Bos taurus* (6); chicken *Gallus gallus domesticus* (20); black duck *Anas rubripes* (3); little corella *Cacatua sanguinea* (12)	Simultaneous infection with Murray Valley encephalitis
Spradbrow [55]	T48	Subcutaneous infection	No	Magnitude and duration of viraemia (LD_50_)	Sheep *Ovis aries* (3); pig *Sus scrofa domesticus* (3)	
Whitehead [38]	T48	Subcutaneous infection	No	Magnitude and duration of viraemia (LD_50_)	Rabbit *Oryctolagus cuniculus* (4); rat *Rattus* spp. (4); bandicoot *Isoodon macrourus* (4); marsupial mouse *Antechinus* spp. (6); chicken *Gallus gallus domesticus* (16); pigeon *Columba livia domestica* (5)	Simultaneous infection with Sindbis virus

*Abbreviations*: SMIC, suckling mouse intracerebral injection; CCID_50_, cell culture infectious dose; TCID, tissue culture infectious dose; LD_50_, lethal dose per gm of whole blood

Comparison of viral titres across experimental infection studies is hampered by different measures of viraemia. Within each study there was substantial variability in the viraemic response reported for different species of animal within each species group and study (i.e. marsupials, placental mammals and birds; Table 3). Whitehead [37] reported the highest peak titres in *Antichinus* spp. of 8 LD_50_ lasting 144 hours, in contrast to 4.75 LD_50_, lasting 48 hours in rabbits (*Oryctolagus cuniculus*). Kay et al. [27] reported the highest viremia in horses (*Equus caballus*) at 6.3 SMIC, compared to black ducks (*Anas rubripes*) developing a peak titre of 1.8 SMIC. Pigeons (*Columba livia domestica*), cats (*Felis catus*) and dogs (*Canis lupus familiaris*) were the only animals that did not develop a detectable viraemia.

**Table 3:** Summary of species, sample size, viraemia and antibody response to experimental infection of vertebrate species with Ross River virus

Species	Reference	Sample size	Proportion with viraemic response	Peak titre level	Viraemia duration (h)	Infected recipient vectors	Proportion with antibody response
Marsupial							
Brushtail possum *Trichosurus vulpecula*	Boyd & Kay [37]	10	0.33	7.5 CCID	48	Yes	0.8
Agile wallaby *Macropus agilis*	Kay et al. [27]	9	0.78	5.6 SMIC	81.6	Not reported	1
Grey kangaroo *Macropus giganteus*	Kay et al. [27]	3	1	4.6 SMIC	144	Not reported	1
Bandicoot *Isoodon macrourus*	Whitehead [38]	4	Not reported	7.2 LD_50_	144	Not reported	1
Marsupial mouse *Antechinus* spp.	Whitehead [38]	6	Not reported	8 LD_50_	144	Not reported	Not reported
Placental mammal							
Horse *Equus caballus*	Kay et al. [27, 34]	11	0.1	6.3 SMIC	96	Yes	0.6
Sheep *Ovis aries*	Kay et al. [27]	8	1	3.8 SMIC	57.6	Not reported	1
	Spradbrow [55]	14	0.64	Not reported	120	Not reported	1
Pig *Sus scrofa domesticus*	Kay et al. [27]	11	0.91	3.0 SMIC	81.6	Not reported	0.45
	Spradbrow [55]	3	1	Not reported	48	Not reported	1
Cow *Bos taurus*	Kay et al. [27]	6	0.16	2.3 SMIC	48	Not reported	0.16
Cat *Felis catus*	Boyd & Kay [58]	10	0	0	0	No	0.1
Dog *Canis lupus familiaris*	Boyd & Kay [58]	10	0	0	0	No	0.1
Grey-headed flying fox *Pteropus poliocephalus*	Ryan et al. [33]	10	0.25	2.2 TCID	Not reported	Yes	0.33
Rat *Rattus* spp*.*	Whitehead [38]	4	Not reported	7.4 LD_50_	72	Not reported	1
Rabbit *Oryctolagus cuniculus*	Kay et al. [27]	9	0.67	3.1 SMIC	55.2	Not reported	Not reported
	Whitehead [38]	4	Not reported	4.7 LD_50_	48	Not reported	1
Bird							
Chicken *Gallus gallus domesticus*	Kay et al. [27]	20	0.95	2.8 SMIC	69.6	Not reported	0.55
	Whitehead [38]	16	Not reported	5.0 LD_50_	120	Not reported	0.75
Black duck *Anas rubripes*	Kay et al. [27]	3	0.67	1.8 SMIC	96	Not reported	1
Little corella *Cacatua sanguinea*	Kay et al. [27]	12	0.5	2.3 SMIC	50.4	Yes	0
Pigeon *Columba livia domestica*	Whitehead [38]	5	0	0	0	Not reported	0.6

*Abbreviations*: SMIC, suckling mouse intracerebral injection; CCID_50_, cell culture infectious dose; TCID, tissue culture infectious dose; LD_50_, lethal dose per gm of whole blood

**Fig. 1:**
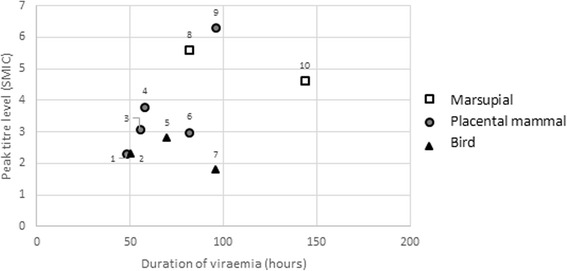
Mean peak titre and duration of viraemia measured in different animals experimentally infected with Ross River virus, data extracted from Kay et al. [27]. Squares represent marsupials, circles represent mammals and triangles represent birds. Species in order of number: 1, Cow; 2, Little corella; 3, Rabbit; 4, Sheep; 5, Chicken; 6, Pig; 7, Black duck; 8, Agile wallaby; 9, Horse; 10, Grey kangaroo

Statistical analysis of the results from Kay et al. [27], the experimental infection study with the greatest number of species (*n* = 10) and largest number of individuals tested (*n* = 92), showed that although viraemia in grey kangaroos (*Macropus giganteus*) attained moderately high levels and lasted the longest duration (Fig. 1), there was no significant difference (between species groups (marsupial (*n* = 11 individuals, 2 species), placental mammals (*n* = 45 individuals, 5 species) and birds (*n* = 35 individuals, 3 species) for both duration of viraemia (*F*_(2, 9)_ = 2.312, *P* = 0.169) and peak titre level (*F*_(2, 9)_ = 3.177, *P* = 0.104).

For horses (*Equus caballus*) and little corellas (*Cacatua sanguinea*), Kay et al. [27] also used susceptible *Cx annulirostris* vectors to feed on infected hosts to determine the percentage of mosquitoes that became infected with RRV. Despite low titre and short duration viraemias (2.3 SMIC, 50 hours; Fig. 1), little corellas infected 14% of an unknown number of recipient vectors. Horses developed the highest titre viraemeia (6.3SMIC), of one of the longest durations (112 hours), and infected a comparable 11% of recipient vectors.

### Virus isolation studies

Isolation of RRV from non-human vertebrate species has been reported in 20 instances in published studies. The majority (*n* = 15) of isolates were recovered from horses (*Equus caballus*), whilst two isolates were recovered from agile wallabies (*Macropus agilis*) and three isolates came from birds (Table 4). Virus was isolated from the heart tissue of birds [38] and from serum of horses and wallabies [28, 39]. Virus isolation was achieved using intracerebral inoculation into infant mice [28, 38] or incubation of tissue culture plates with serum followed by identification with antiserum raised in rabbits [40].

**Table 4:** The number, study and study sample size for isolates of Ross River virus collected from non-human vertebrates

Species	Reference	Sample size	Number of RRV isolations
Marsupial			
Agile wallaby *Macropus agilis*	Doherty et al. [28]	17	2
Mammal			
Horse *Equus callabus*	Azuolas et al. [40]	750	13
	Pascoe et al. [39]	8	1
	Campbell et al. [76]	Not reported	1
Bird			
Magpie lark *Grallina cyanoleuca*	Whitehead et al. [38]	775 (104 species)	1
Flycatcher *Myiagra rubecula*			1
Masked finch *Poephila personata*			1

### Serosurvey studies

**Fig. 2:**
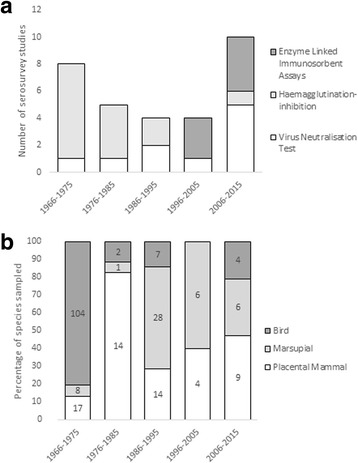
**a** The number and types of method used to test Ross River virus seroprevalence by decade. **b** The percentage of different vertebrate groups sampled in each decade for Ross River virus seroprevalence. The numbers in each bar represent the number of species tested for each species group

We identified a total of 30 serosurveys studies (Additional file 1: Table S2) that tested more than 17,000 serum samples from 77 host species. The majority of these studies were undertaken in Australia, with a small number in New Zealand, Fiji and Papua New Guinea (Table 1). Serosurveys for RRV in non-human species have spanned almost 50 years and, as such, the methods within these studies vary substantially. Studies were grouped by decade of publication to accommodate the different serological methods and species groups tested (Fig. 2). Earlier studies favoured haemoglobin inhibition. This technique has now largely been superseded in favour of assays with better sensitivity and specificity. Virus neutralisation, either through Plaque Reduction Neutralisation (PRNT) (the gold standard) or serum microneutralisation are highly specific [41] and have been used throughout the decades. These methods are generally considered more labour intensive, require trained personnel and a minimum of five days to perform. More recent serosurveys have used enzyme linked immunosorbent assay (ELISA) which can be purchased in commercial kits and are more commonly used in human diagnostic labs (Fig. 2a). In the first decade (1966–1975) of seroprevalence studies, 80% of all species sampled were birds (Fig. 2b). In the following decade (1976–1985) more than 82% of serosurveys were performed on placental mammal species. In the subsequent two decades (1986–2005) marsupial species were sampled most frequently (between 50–60% of serosurveys), followed by placental mammals (between 32–40% of serosurveys) and birds (between 0–17% of serosurveys).

**Fig. 3:**
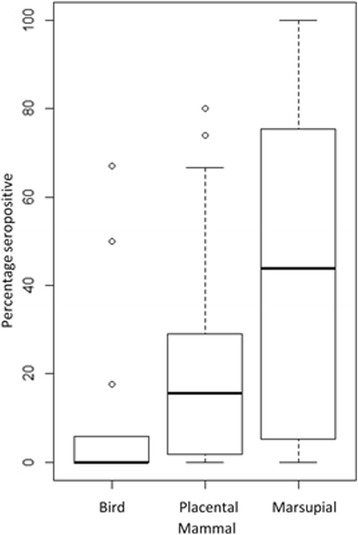
Boxplot of serosurvey results for each vertebrate group with the number of sera sampled in brackets. Minimum, median and maximum values are represented with the box and whiskers, and outliers are represented by circles

Figure 3 shows the mean seropositivity in each of the three species groups. Half of all species sampled were marsupials (*n* = 39 species), followed by placental mammals (*n* = 27 species) and birds (*n* = 13 species). Placental mammals comprised the largest number of sera (*n* = 10,126), more than double that of marsupial sera (*n* = 4304) and quadruple that of bird sera (*n* = 2621). Within placental mammals, cattle have been sampled most frequently (28.9% of samples), closely followed by horses (28.8% of samples). For marsupials, 46% of serosurveys were from one study with a focus on western grey kangaroos, *Macropus fuliginosus* [42]. Within birds, chickens were the most sampled species, contributing 38% of all samples in this species group.

Overall, there was a significant difference in seroprevalence between species groups (*F*_(2, 76)_ = 7.091, *P* = 0.001). Across studies, the median seroprevalence in marsupials was greater when compared with placental mammals and birds (44%, 16% and 0%, respectively; Fig. 3). The interquartile range of seroprevalence was greatest in the marsupial group (5–75%) and smallest in the bird group (0–6%) (Fig. 3). Outliers in the bird seroprevalence results included black ducks (*Anas superciliosa*, 2 of 3 positive) and little corellas (*Cacatua sanguinea*, 6 of 12 positive). For placental mammals, the highest seroprevalence was observed in red foxes (*Vulpes vulpes*, 3 of 4 positive) followed by rabbits (*Oryctolagus cuniculus*, 6 of 10 positive). All of the following marsupial species have tested positive to RRV: the eastern barred bandicoot *Perameles gunnii* (*n* = 2/2), the eastern bettong *Bettongia gaimardi* (*n* = 1/1), the long-nosed potoroo *Potorous tridactylus* (*n* = 2/2), the northern nail-tail wallaby *Onychogalea unguifera* (*n* = 1/1), the Tasmanian devil *Sarcophilus harrisii* (*n* = 4/4) and the tiger quoll *Dasyurus maculatus* (*n* = 1/1).

## Discussion

Identifying reservoirs of multi-host viruses is challenging due to the complex interactions that sustain and promote pathogens and spillover events. For arboviruses, three commonly used criteria for classifying vertebrate reservoirs include: viraemia, virus isolation and relatively high antibody prevalence [3]. In light of these criteria, this study aimed to review the evidence for non-human reservoirs of RRV against the hypothesis: marsupials are better reservoirs of RRV than placental mammals, which are better reservoirs than birds.

### The role of marsupials as reservoirs of RRV

Results from experimental infection, virus isolation and serosurvey studies on 31 marsupial species support the hypothesis that marsupials are competent reservoirs and likely contribute significantly to RRV transmission. However, the evidence is fragmentary and subject to sampling bias, which limits our ability to extrapolate across species, broad geographical areas, habitat and land use types.

Across experimental infection studies, marsupials generally developed high and long-lasting viraemia. This has previously been interpreted as evidence that marsupials are better reservoirs than other species groups, yet we found no significant difference between the mean duration of viraemia or peak viraemia of marsupials, placental mammals or birds. At least two factors must be considered when interpreting results of experimental infection studies. First, experimental infection studies are often constrained by small sample sizes both in the number of species and the number of studies that can be compared. Although we statistically analysed results from the RRV experimental infection study with the greatest number and diversity of species [27], sample sizes were still limited and likely influenced the statistical power of the results. In particular, the diversity of methods used limits comparisons and the effect of simultaneous co-infection with other viruses cannot be discounted. Secondly, while viraemia plays an important role in the maintenance and transmission of arboviruses, using this measure alone to identify potential reservoirs has limited value. For example, in experimental infection studies of West Nile virus, another zoonotic arbovirus, viraemia alone did not definitively identify vertebrate reservoirs: blue jays (*Cyanocitta cristata*), house finches (*Haemorhous mexicanus*) and house sparrows (*Passer domesticus*) were identified as the most competent reservoirs on the basis of viraemia profile [43]. Yet subsequent field investigations identified that American robins (*Turdus migratorius*), a less viraemic and relatively uncommon avian species, were responsible for the majority of WNV vector infections due to host feeding preferences [44].

The isolation of virus from naturally infected hosts is interpreted as evidence that the species can infect vector mosquitoes and, thus, infect humans. For marsupials, the isolation of RRV from two free-living agile wallabies (*M. agilis*) (from a total of 17 tested) demonstrates a vector-host relationship under natural conditions and suggests that this species is capable of infecting susceptible vectors, thereby supporting the argument for the species as reservoirs of RRV. Together with Kay et al.’s [27] observation of viraemia in grey kangaroos, this has led to the hypothesis that macropods are important RRV reservoirs within their range. However, the relative importance of this group of species as a reservoir is not clear, given that RRV has been isolated more frequently from horses and passerine birds and the majority of RRV cases in humans do not overlap with macropod home ranges [13].

Across all studies, marsupials had the highest RRV seroprevalence (44.3%), compared with placental mammals (22.7%) and birds (11.1%). Although informative, these data must be interpreted with caution because it is evident that marsupials were more likely to be targeted during sampling efforts in the decade 1986–1995 (Fig. 2b). This shift in targeted species group followed the results of experimental infection studies demonstrating marsupials as competent amplifiers of RRV in 1986. Further, without information on the age of individuals, seroprevalence data should be compared between studies with caution.

Sampling biases are likely to have arisen from the frequent use of convenience sampling or ‘active surveillance’ methods (where investigator-driven data collection is designed to meet specific information needs [45]). The focus on marsupials as hosts for RRV to the exclusion of other host species is premature, and is unlikely to be uniform across all marsupial species. For example, brushtail possums were hypothesised to be the urban reservoir of RRV, being both marsupials and living in close proximity to humans [35], resulting in a focus on this species. However, targeted surveillance of this species between February and December 2005 in Sydney failed to identify any seropositive individuals [35, 46] of the 10 possums sampled. This number of animals is insufficient to draw strong conclusions about the host status, and further studies are required [46]. Furthermore, it is interesting to note that whilst brushtail possums are an abundant urban marsupial, ringtail possums are more commonly reported in major metropolitan areas including Brisbane, Sydney, Perth, Adelaide and Hobart [47] but only two studies (testing four individuals in total) have been undertaken serological assessments of the species (50% seropositivity) [48, 49].

### The role of placental mammals as reservoirs of RRV

Placental mammals comprise the greatest diversity of species tested, including ungulates, carnivorous and small urban species. While placental mammals meet the three criteria for arboviral reservoirs as a species group, there are significant differences among species.

#### Ungulates

Ungulate species, including pigs, horses, sheep and cattle, are recognised as reservoirs for other zoonotic arboviruses [4]. Interestingly for RRV, horses are the only ungulate likely to amplify the disease and act as reservoirs. High, long-lasting viral titres, the ability to infect susceptible mosquitoes, frequent virus isolations and high seroprevalences suggest that horses could contribute significantly to ongoing RRV transmission, particularly during epidemic periods [50], although it is unclear whether they play a role in ongoing endemic circulation of RRV. A possible explanation for the high number of RRV isolates from horses is that they are both a domestic species and one of the only known species that develops clinical symptoms to RRV [51] and therefore, are more likely to be sampled, particularly if they were infected and symptomatic. The horse population in Australia may exceed 1.2 million individuals [52], and whilst they are rare in highly urbanised environments, they are abundant in peri-urban areas, where some of the highest prevalence of human RRV infection exists [53].

In contrast, RRV has not been isolated from cattle, pigs and sheep [54–56], and these species have demonstrated low viraemic responses in experimental infection studies [27] and in serosurveys [55]. Large numbers of cattle sera are tested for antibodies to RRV due to the use of cattle as sentinel species in the National Arbovirus Monitoring Program, which is designed to detect incursions of exotic arboviral infection, such as bluetongue viruses [57].

#### Cats and dogs

Cats and dogs are the only carnivores that have been assessed as potential reservoir hosts of RRV. Viraemias were not detected following experimental infection and only 10% of these cats and dogs developed neutralising antibodies to RRV [58]. Seroprevalence studies of domestic cats not experimentally infected, found they have a relatively low antibody prevalence (12.1%). The poor amplifier capacity and low seroprevalence suggest these domestic species are unlikely to be significant reservoirs of RRV.

#### Small mammals (< 2 kg)

The potential role of small placental mammals, such as rodents, rabbits and flying foxes, as reservoir hosts of RRV is ambiguous. Under experimental infection conditions Whitehead [37] found rodents were capable of developing viraemia higher than bandicoots, a marsupial, yet the viraemia was short lived compared to marsupials. Rabbits developed mid-range titre peaks of short duration.

In experimental infection, grey-headed flying foxes (*Pteropus poliocephalus*) did not develop a detectable viraemia, but were capable of infecting 3% of recipient *Ae. vigilax* vectors [33]. Flying foxes are a unique species group because they have been shown to be the reservoir host for several zoonotic pathogens including, henipaviruses lyssaviruses and filoviruses, often without detectable viraemia [6, 59]. Similar observations have been made for arboviruses. In an experimental infection of black flying foxes (*Pt. alecto*) with Japanese encephalitis virus, all 15 individuals had a low viraemic response; however, two were capable of infecting susceptible mosquitoes [60]. Only the grey-headed flying fox has been investigated as a potential reservoir host of RRV, yet a blood meal analysis of 20 *Ae. funereus* vectors in close proximity to a mixed-species flying fox colony in Brisbane found that all of the 16 mosquitoes analysed had fed on black flying foxes and none on grey-headed flying foxes [33]. When considering the possibility of flying foxes as reservoirs of RRV, it is important to consider the height at which different vectors feed and move. Known vectors of RRV, including *Ae. vigilax* and *Ae*. *camptorhyncus* are likely to feed close to the ground, potentially avoiding roosting flying foxes [61]. Further blood meal analysis studies are needed to determine this.

Given their small body size, rats, rodents and flying foxes may be considered less desirable as blood-meals for vectors [62]. However, they may exist in high densities close to human populations. A blood-meal analysis of RRV vectors found rabbits and rats comprised up to 33% of *Cx annulostris* blood-meals in urban areas [63]. Serological data supporting the hypothesis that small mammals may be playing a role in the transmission of RRV is currently lacking due to limited numbers tested.

### The role of birds as reservoirs of RRV

Birds are the most common arboviral reservoir for zoonotic flaviviruses and alphaviruses globally [4]; however, their contribution as reservoirs of RRV has been largely overlooked. On the basis of experimental infection viraemia data alone, birds appear to be poor amplifiers of RRV. Four species of birds (chickens, pigeons, little corellas and black ducks) have been experimentally infected with RRV. Across experimental infection studies, birds had the lowest peak titre and the shortest duration of viraemia in comparison to marsupials and placental mammals (Table 3). Furthermore, pigeons were one of the only species that did not develop a detectable viraemia. However, little corellas (*Cactua sanguinea*) were capable of infecting 14% of susceptible *Cx annuilostris* mosquitoes, despite having a low and short viraemia. This is important because in the same study, horses developed the highest titre but only infected 11% of susceptible vectors. Possible reasons for this were not discussed in the original paper, but we suggest the capability of a vertebrate species to infect susceptible mosquito vectors with RRV may be a more relevant measure of reservoir capacity than viraemia.

The isolation of RRV from birds further supports their capacity as amplifiers. More than 750 virus isolation attempts, across 104 species, yielded the first 3 isolates of RRV from the heart muscle of passerine birds in Northern Queensland: a magpie lark, a flycatcher and a masked finch (Table 4). Passerine birds are recognised as important amplifiers of other arboviruses including flaviviruses such as West Nile virus [43], tick-borne pathogens such as *Borrelia burgdorferi* - the causative agent for Lyme disease [64] and an arthritic alphavirus closely related to RRV, Sindbis [65]. Indeed, the isolation of the alphavirus Sindbis from passerine birds, in combination with genetic studies and antibody prevalence investigations has implicated birds as the reservoir host of Sindbis [62].

Serological surveys have found low seroprevalence of RRV in birds. However almost 40% of bird sera tested has been from sentinel chickens. Chickens are considered appropriate sentinels for flaviviruses such as Murray valley encephalitis because they display a strong antibody response [66], however experimental infections suggest this is not the case for RRV [27, 37]. Notably, birds with positive serology for RRV were free-living native species: a Tawny frogmouth owl in NSW [48] and an Australasian gannet (*Morus serrator*) sampled in New Zealand [67]. Thus, the tendency towards sampling chickens in RRV serosurveys may underestimate the rates for birds as a whole, and future serosurveys would benefit from inclusion of greater bird species diversity.

### Alternative evidence for non-human reservoirs

This review has focused on the intrinsic host variables important to reservoir capacity. There are other lines of evidence that can be important for investigating potential reservoirs such as blood meal analysis and modelling studies. Determining vector preferences, may indicate a higher feeding frequency, and thus if a capable reservoir, higher transmission rate. Blood meal analysis studies investigate the relationship between the vector and the host. Vector-host choice is a complicated matter, with factors such as host body size, carbon dioxide emission, olfaction, availability, abundance and vector genetics impacting feeding preferences [62, 68]. Blood meal studies are further complicated as they are easily confounded by the environment in which study took place, and as such these studies are best when accompanied with animal abundance and diversity measures. Of the 12 blood meal analysis papers in Australia, only one [69] has done this by asking the human residents to estimate numbers of animals. Further research is needed to investigate vector-host preferences in Australian urban, peri-urban and rural environments and determine the influence this may have on a species capacity to act as a reservoir.

Mathematical models are valuable way of describing and understanding complex disease systems such as RRV. Models can test assumptions of a disease system and generate predictions which can be used for management decisions. For RRV, five studies [20, 70–73] have utilised mechanistic modelling techniques (e.g. Susceptible-Infectious-Recovered models) to better understand the transmission dynamics underpinning the maintenance of the pathogen. Although the models differ in parameters, location and methods, all include a marsupial reservoir. Species that have been modelled as reservoirs are western grey kangaroos and brushtail possums. Overall these studies found that one host alone was insufficient to maintain virus in vector populations. Glass [72] concluded that although marsupials such as kangaroos and wallabies are generally assumed to be the most important reservoir hosts, the virus survived longed under all models when the marsupial host was replaced with one with a shorter infectious period and higher birth rate. Further, the same study reported that very large host populations (> 100,000 individuals) were required for the virus to survive for four years. Choi et. al. [70] similarly found that a kangaroo reservoir did not impact the number of human infections due to a small population size in the region. Carver et al. [20] reported a significant negative relationship between the abundance of a marsupial reservoir and RRV transmission. These findings are in contrast to the putative reservoir hypothesis. Tompkins & Slaney [73] noted that different species may be reservoirs in different environments, such as high-density urban areas and protected environmental habitats, which can result in different transmission cycles. These modelling studies highlight the importance of investigating alternative species as potential reservoirs of RRV. Ideally, the system should be explicitly modelled as a multihost system, but obtaining the necessary data to parameterise such models is challenging [74].

### Ross River virus: a multi-host pathogen

Despite the evidence supporting marsupials as reservoirs of RRV, questions remain. Recent studies have found a high seroprevalence of RRV in the Pacific Islands in the absence of marsupial populations [29, 31], suggesting that marsupials are not the only species group capable of increasing the community infection for RRV. Studies modelling RRV reservoirs have suggested that the pathogen has a multi-host system [23, 72]. However, none of the studies reviewed in this paper specifically examined this hypothesis. Multi-host systems are not uncommon for arboviruses but quantifying these systems is challenging, requiring coordinated data collection over temporal and geographical scales for multiple species [3].

To understand RRV as a multi-host pathogen, two issues must be considered. First, as RRV has an international distribution spanning different environmental and social bounds it is important to define the ecological transmission of RRV across different ecosystems. Expansion of Claflin & Webb’s [14] categorisation of potential RRV vectors, habitats and reservoirs across inland, metropolitan and coastal regions to include transmission cycles is warranted. Secondly, to better understand the reservoir capacity between different host communities, identification of amplifying or diluting reservoir hosts is required. Given that humans are not considered significant maintenance reservoirs of RRV outside of epidemic periods in Australia, this may provide a benchmark for relative comparison of seroprevalence. For example, RRV seroprevalence in blood donors shows that the human IgG seroprevalence ranges from 8.38% in Australia in 2011 [75] to 34.4% in French Polynesia between 2011 and 2013 [29]. Whilst this only gives an indication of the number of people exposed and does not consider other contributing factors such as duration of antibody response, these data may be compared with animal serosurvey data to identify species with higher infection rates than humans. Vector-host preference may be key to understanding reservoir and transmission dynamics in other zoonotic arboviruses [3]. Overall, consideration of RRV as a multi-host pathogen may disentangle the complex ecological dynamics that may be taking place.

## Conclusion

This study set out to: (i) critically review the evidence for the hypothesis that marsupials are better reservoirs of RRV than mammals, which in turn are better than birds; (ii) identify limitations of this evidence; and (iii) identify research gaps allowing for better assessments of RRV reservoirs in the future. The evidence reviewed in this paper is limited by a sampling bias in favour of particular species and species groups, cross-sectional serosurveys and a diversity of methods employed, which reduces the statistical strength for meta-analysis. Notwithstanding these limitations, this review highlights that evidence to support the stated hypothesis, that marsupials are better reservoirs than placental mammals which in turn are better reservoirs than birds, is variable. Understanding the non-human reservoirs of RRV has broader applications to other zoonotic arboviruses and, importantly, can contribute to the management of current and emerging arboviruses through mitigating infection between host and vector populations. Future research on the non-human reservoirs of RRV should focus on investigating non-marsupial species, including passerine birds and small placental mammals. Ideally this would be done through ecological assessments of vector, virus and host abundance in areas of high and low disease in humans. For Australia, reducing the burden of RRV, the most common arbovirus, would have substantial economic and social benefits.

## Additional files

Additional file 1: **Table S1**. Combinations of search terms used to collect papers for review. **Table S2**. Detailed summary of included Ross River virus reservoir studies, including the reference, location, study type and species group assessed in each study. (DOCX 22 kb)

Additional file 2: **Figure S1**. Flowchart outlining the process followed and actions taken to compile the systematic literature review. The box in yellow highlights the total number of studies used in this review. The total number *n* is the number of original research papers. (PNG 29 kb)

## Abbreviations

ANOVA: Analysis of Variance
ELISA: Enzyme linked immunosorbent assay
PRNT: Plaque reduction neutralisation
RRV: Ross River virus
