# Supplementary material for: The non-human reservoirs of Ross River virus: a systematic review of the evidence
Source: Parasit Vectors. 2018 Mar 19;11:188. doi: 10.1186/s13071-018-2733-8 (PMC5859426; doi:10.1186/s13071-018-2733-8)
Supplement: Supplementary file 1 — Table S1. Combinations of search terms used to collect papers for review. Table S2. Detailed summary of included Ross River virus reservoir studies, including the reference, location, study type and species group assessed in each study. (DOCX 22 kb) [file 13071_2018_2733_MOESM1_ESM.docx]

**Additional file 1: Table S1** Combinations of search terms used to collect papers for review.

| **Search term 1 (virus related)** | **Combined with the following text relating to animals** | **Combined with the following text related to testing methods** |
| --- | --- | --- |
| Ross River virus | AND; host OR reservoir OR wild* OR captive OR population | AND serolog* OR serosurvey* OR antibod*OR virus OR viral OR viraemia OR viremia OR PCR OR patholog* OR serum OR RNA OR vector* |
| Ross River fever |  |  |
| Epidemic polyarthritis |  |  |

The asterisk (*) operator was used as a wildcard to search for all the possible variations of keywords.

**Additional file 1: Table S2** Detailed summary of included Ross River virus reservoir studies, including the reference, location, study type and species group assessed in each study.

|  | **Reference** | **Location** | **Study type** | **Species group** |
| --- | --- | --- | --- | --- |
| 1 | Doherty, R. L., Gorman, B. M., Whitehead, R. H., & Carley, J. G. (1966). Studies of arthropod-borne virus infections in Queensland .V. Survey of antibodies to Group A arboviruses in man and other animals *Australian Journal of Experimental Biology and Medical Science, 44*, 365-&. doi:10.1038/icb.1966.35 | Queensland | Serosurvey | Marsupial/Placental mammal/Bird |
| 2 | Whitehead, R., Doherty, R., Domrow, R., Standfast, H., & Wetters, E. (1968). Studies of the epidemiology of arthropod-borne virus infections at Mitchell River Mission, Cape York Peninsula, North Queensland: III. Virus studies of wild birds, 1964–1967. *Transactions of the Royal Society of Tropical Medicine and Hygiene, 62*(3), 439-445. | Queensland | Virus isolation/Serosurvey | Bird |
| 3 | Whitehead, R. (1969). Experimental infection of vertebrates with Ross River and Sindbis viruses 2 Group A arboviruses isolated in Australia. *Australian Journal of Experimental Biology and Medical Science, 47*, 11-&. doi:10.1038/icb.1969.2 | Queensland | Experimental infection | Marsupial/Placental mammal/Bird |
| 4 | Sanderson, C. (1969). A serologic survey of Queensland cattle for evidence of arbovirus infections. *The American Journal Of Tropical Medicine And Hygiene, 18*(3), 433. | Queensland | Serosurvey | Placental mammal |
| 5 | Doherty, R. L., Standfast, H. A., Domrow, R., Wetters, E. J., Whitehead, R. H., & Carley, J. G. (1971). Epidemiology of arthropod-borne virus infections at Mitchell River Mission, Cape York Peninsula, North Queensland. IV. Arbovirus infections of mosquitoes and mammals, 1967-1969. *Transactions of the Royal Society of Tropical Medicine and Hygiene, 65*(4), 504-&. doi:10.1016/0035-9203(71)90161-1 | Queensland | Virus isolation/Serosurvey | Marsupial/Placental mammal/Bird |
| 6 | Spradbrow, P. B. (1972). A survey for arbovirus antibodies in pigs and sheep in Queensland. *Australian Veterinary Journal, 48*(7), 402-407. | Queensland | Serosurvey | Placental mammal |
| 7 | Chung, Y. S., & Spradbrow, P. B. (1973). Survey for antibodies to arboviruses in domestic-fowls in Queensland. *Australian Veterinary Journal, 49*(12), 564-&. doi:10.1111/j.1751-0813.1973.tb06734.x | Queensland | Serosurvey | Placental mammal |
| 8 | Doherty, R. L., George, T. D. S., & Carley, J. G. (1973). Arbovirus infections of sentinel cattlel in Australia and New-Guinea. Australian Veterinary Journal, 49(12), 574-579. doi:10.1111/j.1751-0813.1973.tb06737.x | Queensland, Western Australia, Northern Territory, New South Wales, New Guinea | Serosurvey | Placental mammal |
| 9 | Gard, G., Marshall, I. D., & Woodroof, G. M. (1973). Annually recurrent epidemic polyarthritis and Ross River virus activity in a coastal area of New South Wales. II. Mosquitos, viruses and wildlife. *American Journal of Tropical Medicine and Hygiene, 22*(4), 551-560. | New South Wales | Serosurvey | Marsupial/Placental mammal/Bird |
| 10 | Spradbrow, P. B. (1973). Experimenatl infection of sheep and pigs with Ross River virus. *Australian Veterinary Journal, 49*(8), 403-404. doi:10.1111/j.1751-0813.1973.tb09357.x | Queensland | Experimental infection | Placental mammal |
| 11 | Gard, G. P., Giles, J. R., Dwyergray, R. J., & Woodroofe, G. M. (1976). Serological evidence of inter-epidemic infection of feral pigs in New South Wales with Murray Valley encephalitis virus. *Australian Journal of Experimental Biology and Medical Science, 54*(JUN), 297-302. doi:10.1038/icb.1976.30 | New South Wales | Serosurvey | Marsupial/Placental mammal/Bird |
| 12 | Pascoe, R., George, T., & Cybinski, D. (1978). The isolation of a Ross River virus from a horse. *Australian Veterinary Journal, 54*(12), 600-600. | Queensland | Virus isolation | Placental mammal |
| 13 | Marshall, I. D., Woodroofe, G. M., & Gard, G. P. (1980). Arboviruses of coastal Southeastern Australia. *Australian Journal of Experimental Biology and Medical Science, 58*(Pt1), 91-102. doi:10.1038/icb.1980.9 | New South Wales | Sersosurvey | Marsupial/Placental mammal |
| 14 | Cloonan, M. J., O'Neill, B. J., Vale, T. G., Carter, I. W., & Williams, J. E. (1982). Ross River virus activity along the south coast of New South Wales. *Australian Journal of Experimental Biology and Medical Science, 60*(DEC), 701-706. doi:10.1038/icb.1982.71 | New South Wales | Serosurvey | Placental mammal |
| 15 | Marshall, I. D., Brown, B. K., Keith, K., Gard, G. P., & Thibos, E. (1982). Variation in arbovirus infection rates in species of birds sampled in a serological survey during an encephalitis epidemic in the Murray-Valley of Southeastern Australia, February 1974. *Australian Journal of Experimental Biology and Medical Science, 60*(Pt 5), 471-478. doi:10.1038/icb.1982.52 | Western Australia | Serosurvey | Placental mammal |
| 16 | Marshall, I., & Miles, J. (1984). Ross River virus and epidemic polyarthritis. *Curr Top Vector Res, 2*, 31-56. | Fiji | Serosurvey | Placental mammal |
| 17 | Kay, B. H., Hall, R. A., Fanning, I. D., Mottram, P., Young, P. L., & Pollitt, C. C. (1986). Experimental infection of vertebrates with Murray Valley encephalitis and Ross River viruses. *Arbovirus Research in Australia, 1986*, 71-75. | Queensland | Experimental infection | Marsupial/Placental mammal /Bird |
| 18 | McManus, T. J., & Marshall, I. D. (1986). The epidemiology of Ross River virus in Tasmania. *Arbovirus Research in Australia, 1986*, 127-131. | Tasmaina | Serosurvey | Marsupial/Placental mammal |
| 19 | O'Callaghan, M. G., & Moore, E. (1986). Parasites and serological survey of the common brushtail possum (Trichosurus vulpecula) from Kangaroo Island, South Australia. *Journal of Wildlife Diseases, 22*(4), 589-591. | South Australia | Serosurvey | Marsupial/Placental mammal |
| 20 | Kay, B. H., Pollitt, C. C., Fanning, I. D., & Hall, R. A. (1987). The experimental infection of horses with Murray Valley encephalitis and Ross River viruses. *Australian Veterinary Journal, 64*(2), 52-55 | Queensland | Experimental infection | Placental mammal |
| 21 | Campbell, J., Aldred, J., & Davis, G. (1989). *Some aspects of the natural history of Ross River virus in south east Gippsland, Victoria.* Paper presented at the Arbovirus research in Australia. Proceedings Fifth Symposium, August 28-September 1, 1989, Brisbane, Australia. | Victoria | Serosurvey/Virus isolation | Placental mammal/Marsupial |
| 22 | Aldred, J., Campbell, J., Mitchell, P., Davis, G., & Elliott, J. (1991). *Involvement of wildlife in the natural cycles of Ross River and Barmah Forest viruses.* Paper presented at the Proceedings of the Wildlife Disease Association (Australasian Section) Annual Conference, Malacoota. | Victoria | Serosurvey | Marsupial/Placental mammal |
| 23 | Humphery-Smith, I., Cybinski, D. H., Byrnes, K. A., & George, T. D. S. (1991). Seroepidemiology of arboviruses among seabirds and island residents of the Great Barrier Reef and Coral Sea. *Epidemiology and Infection, 107*(2), 435-440. | Queensland | Serosurvey | Birds |
| 24 | Vale, T. G., Spratt, D. M., & Cloonan, M. J. (1991). Serological evidence of arbovirus infection in native and domesticated mammals on the south coast of New South Wales Australia. *Australian Journal of Zoology, 39*(1), 1-8. | New South Wales | Serosurvey | Marsupial/Placental mammal |
| 25 | Azuolas, J. (1997). Arboviral diseases of horses and possums. *Arbovirus Res. Aust, 7*, 5-7. | Victoria | Serosurvey | Marsupial/Placental mammal |
| 26 | Ryan, P. A., Martin, L., Mackenzie, J. S., & Kay, B. H. (1997). Investigation of gray-headed flying foxes (Pteropus poliocephalus) (Megachiroptera: Pteropodidae) and mosquitoes in the ecology of Ross river virus in Australia. *American Journal of Tropical Medicine and Hygiene, 57*(4), 476-482. | Queensland | Experimental infection | Placental mammal |
| 27 | Azuolas, J. K. (1998). Ross River virus disease of horses. *Australian Equine Veterinarian, 16*(2), 56-58. | Victoria | Serosurvey | Placental mammal |
| 28 | Boyd, A. M., Hall, R. A., Gemmell, R. T., & Kay, B. H. (2001). Experimental infection of Australian brushtail possums, Trichosurus vulpecula (Phalangeridae : Marsupialia), with Ross River and Barmah Forest viruses by use of a natural mosquito vector system. *American Journal of Tropical Medicine and Hygiene, 65*(6), 777-782. | Queensland | Experimental infection | Marsupial |
| 29 | Boyd, A. M., & Kay, B. H. (2002). Assessment of the potential of dogs and cats as urban reservoirs of Ross River and Barmah Forest viruses. *Australian Veterinary Journal, 80*(1-2), 83-86. | Queensland | Experimental infection | Placental mammal |
| 30 | Azuolas, J. K., Wishart, E., Bibby, S., & Ainsworth, C. (2003). Isolation of Ross River virus from mosquitoes and from horses with signs of musculo-skeletal disease. *Australian Veterinary Journal, 81*(6), 344-347. doi:10.1111/j.1751-0813.2003.tb11511.x | Victoria | Virus isolation | Placental mammal |
| 31 | Old, J. M., & Deane, E. M. (2005). Antibodies to the Ross River virus in captive marsupials in urban areas of eastern New South Wales, Australia. *Journal of Wildlife Diseases, 41*(3), 611-614. | New South Wales | Serosurvey | Marsupial |
| 32 | Kay, B. H., Boyd, A. M., Ryan, P. A., & Hall, R. A. (2007). Mosquito feeding patterns and natural infection of vertebrates with Ross River and Barmah Forest viruses in Brisbane, Australia. *American Journal of Tropical Medicine and Hygiene, 76*(3), 417-423. | Queensland | Serosurvey | Marsupial/Placental mammal |
| 33 | Hill, N. J., Power, M. L., & Deane, E. M. (2009). Absence of Ross River virus amongst Common brushtail possums (Trichosurus vulpecula) from metropolitan Sydney, Australia. *European Journal of Wildlife Research, 55*(3), 313-316. doi:10.1007/s10344-008-0238-z | New South Wales | Serosurvey | Marsupial |
| 34 | McFadden, A. M. J., McFadden, B. D., Mackereth, G. F., Clough, R. R., Hueston, L., Gradwell, B., & Dymond, M. (2009). A serological survey of cattle in the Thames-Coromandel district of New Zealand for antibodies to Ross River virus. *New Zealand Veterinary Journal, 57*(2), 116-120. doi:10.1080/00480169.2009.36888 | New Zealand | Serosurvey | Placental mammal |
| 35 | Pacioni, C., Johansen, C. A., Mahony, T. J., O'Dea, M. A., Robertson, I. D., Wayne, A. F., & Ellis, T. (2013). A virological investigation into declining woylie populations. *Australian Journal of Zoology, 61*(6), 446-453. doi:10.1071/zo13077 | Western Australia | Serosurvey | Marsupial |
| 36 | Tompkins, D., Johansen, C., Jakob-Hoff, R., Pulford, D., Castro, I., & Mackereth, G. (2013). Surveillance for arboviral zoonoses in New Zealand birds. *Western Pacific surveillance and response journal : WPSAR, 4*(4), 16-23. doi:10.5365/wpsar.2013.4.3.002 | New Zealand | Serosurvey | Bird |
| 37 | Potter, A., Johansen, C. A., Fenwick, S., Reid, S. A., & Lindsay, M. D. A. (2014). The seroprevalence and factors associated with Ross River virus infection in Western grey kangaroos (Macropus fuliginosus) in Western Australia. *Vector-Borne and Zoonotic Diseases, 14*(10), 740-745. doi:10.1089/vbz.2014.1617 | Western Australia | Serosurvey | Placental mammal |
| 38 | Reiss, A., Jackson, B., Gillespie, G., Stokeld, D., & Warren, K. (2015). *Investigation of Potential Diseases Associated with Northern Territory Mammal Declines*. | Northern Territory | Serosurvey | Marsupial/Placental mammal |
