# Supplementary figures and images for: The non-human reservoirs of Ross River virus: a systematic review of the evidence
Source: Parasit Vectors. 2018 Mar 19;11:188. doi: 10.1186/s13071-018-2733-8 (PMC5859426; doi:10.1186/s13071-018-2733-8)

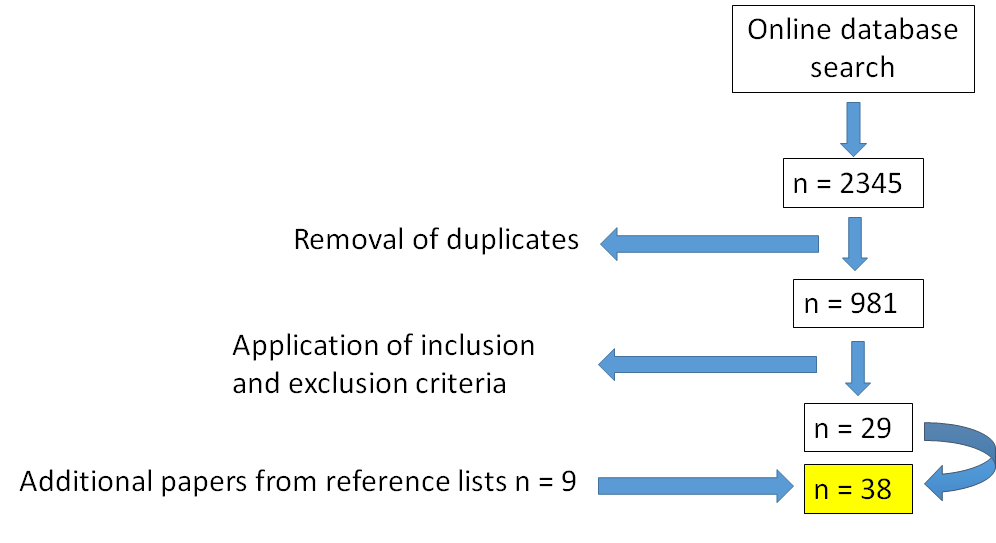

Supplement: Supplementary file 2 — Figure S1. Flowchart outlining the process followed and actions taken to compile the systematic literature review. The box in yellow highlights the total number of studies used in this review. The total number n is the number of original research papers. (PNG 29 kb) [file 13071_2018_2733_MOESM2_ESM.png]
